# Preparations of Meiotic Pachytene Chromosomes and Extended DNA Fibers from Cotton Suitable for Fluorescence *In Situ* Hybridization

**DOI:** 10.1371/journal.pone.0033847

**Published:** 2012-03-19

**Authors:** Renhai Peng, Tao Zhang, Fang Liu, Jian Ling, Chunying Wang, Shaohui Li, Xiangdi Zhang, Yuhong Wang, Kunbo Wang

**Affiliations:** 1 State Key Laboratory of Cotton Biology, China and Cotton Research Institute of Chinese Academy of Agricultural Science, Anyang, Henan, China; 2 Anyang Institute of Technology, Anyang, China; 3 Cash Crop Research Institute, Hubei Academy of Agricultural Sciences, Wuhan, China; East Carolina University, United States of America

## Abstract

Fluorescence *in situ* hybridization (FISH) has become one of the most important techniques applied in plant molecular cytogenetics. However, the application of this technique in cotton has lagged behind because of difficulties in chromosome preparation. The focus of this article was FISH performed not only on cotton pachytene chromosomes, but also on cotton extended DNA fibers. The cotton pollen mother cells (PMCs) instead of buds or anthers were directly digested in enzyme to completely breakdown the cell wall. Before the routine acetic acid treatment, PMCs were incubated in acetic acid and enzyme mixture to remove the cytoplasm and clear the background. The method of ice-cold Carnoy's solution spreading chromosome was adopted instead of nitrogen removed method to avoid chromosomes losing and fully stretch chromosome. With the above-improved steps, the high-quality well-differentiated pachytene chromosomes with clear background were obtained. FISH results demonstrated that a mature protocol of cotton pachytene chromosomes preparation was presented. Intact and no debris cotton nuclei were obtained by chopping from etiolation cotyledons instead of the conventional liquid nitrogen grinding method. After incubating the nuclei with nucleus lysis buffer on slide, the parallel and clear background DNA fibers were acquired along the slide. This method overcomes the twist, accumulation and fracture of DNA fibers compared with other methods. The entire process of DNA fibers preparation requires only 30 min, in contrast, it takes 3 h with routine nitrogen grinding method. The poisonous mercaptoethanol in nucleus lysis buffer is replaced by nonpoisonous dithiothreitol. PVP40 in nucleus isolation buffer is used to prevent oxidation. The probability of success in isolating nuclei for DNA fiber preparation is almost 100% tested with this method in cotton. So a rapid, safe, and efficient method for the preparation of cotton extended DNA fibers suitable for FISH was established.

## Introduction

Fluorescence *in situ* hybridization (FISH) allows direct mapping of DNA sequences on chromosomes and has become an important technique in plant molecular cytogenetics research such as the detection of alien chromosomes [1]–[4] and mapping of small low-copy DNA sequences in various species [5], [6]. One of the most important applications for FISH is physical mapping in plant [7]–[12]. FISH-based physical mapping provides a valuable complementary approach in genome sequencing, such as measuring the physical distances between adjacent BAC contigs [13]–[15] and delineating the structure and DNA composition of genomic regions of centromere and telomere [16], [17]. The resolution of FISH-based physical mapping depends on the condensed degree of target DNA. For example, mitotic metaphase chromosomes are good target DNA for FISH but too condensed for high-resolution physical mapping. So researchers are engaged in resolving FISH signals of two DNA clones to a distance of several MBs [18]. To enhance FISH resolution, meiotic pachytene chromosomes and extended DNA fibers have been developed in some plants, such as *Arabidopsis*, tomato and rice, etc. [13], [14], [17], [19]–[25]. The FISH resolution of pachytene chromosomes could reach 100 KBs [26] and even be used to detect partially overlapped BAC clones [27]. FISH on extended DNA fibers has greatly improved the resolution and sensitivity of DNA sequences, which has reached a resolution of several KBs [28], [29]. The method has been extensively applied to measure the length and copy numbers of repetitive sequences of genome [30] and to determine the physical distance between genes [31], and it also has been used to physically map BAC or YAC contigs with high resolution [32], [33] and to analyze gap size in physical mapping [14], [15].

Cotton is the leading natural fiber crop in the world. Its genomics studies have been developed rapidly and even its genomic sequencing is under way [34]–[36]. The cotton genus *Gossypium* is composed of 45 dipoid species (*n* = *x* = 13) which fall into eight different genome groups designated as A through G, and K, based on meiotic pairing behavior and 5 tetraploid species (*n* = 2*x* = 26) designated AD genome group. Among them only 4 are cultivated species: *G. hirsutum* L. (AD)_1_, *G. barbadense* L. (AD)_2_, *G. herbaceum* (A)_1_, and *G. arboreum* (A)_2_. It was believed that the tetraploid cotton originated from an interspecific hybridization of an Old World diploid species that was closely related with *G. arboreum* or *G. herbaceum* (A genome donor) and a New World diploid species relative to *G. raimondii* Ulbrich or *G. gossipioide*s Standley (D genome donor), which occurred about 1∼2 million years ago [37]–[40]. *G. arboreum*, generally regarded as one of the best exemplars of the A-subgenome progenitors, has been domesticated and cultivated in China for almost 2000 years [41], [42]. Due to some of its superior agronomic traits, such as higher fiber strength, disease and insect resistance, and excellent plasticity, which upland cotton cultivars lack, *G. arboreum* is still planted and is used worldwide as a germplasm resource in present-day cotton breeding programs. Therefore, the *G. arboreum* species is important for genomic and evolution research in cotton, especially comparative genome sequencing in cotton. FISH plays an important role in the methods of cotton complete sequencing. FISH target DNA in cotton used to be mitotic and meiotic metaphase chromosomes [10]–[12], [43]–[47]. Recently, FISH studies on tetraploid cotton pachytene chromosomes have been reported originally [48]. But the well-differentiated pachytene chromosomes can't be easily obtained, and FISH on extended DNA fibers has not been reported yet, due to the large number of chromosomes, thick organic constituent and cytoplasm, and hard cell wall [43]–[45], [48], which lead to the difficulties in pachytene chromosomes and DNA fibers preparation. It is necessary to develop a protocol for preparing meiotic pachytene chromosomes and extended DNA fibers in cotton. In this study, we presented a highly efficient preparation method in cotton to develop pachytene chromosomes and extended DNA fibers hybridized with telomere probe, 45S rDNA probe and genomic DNA probe, respectively.

## Materials and Methods

### Plant materials

CRIGA-1, a genetic stock of diploid cotton [*Gossypium arboreum* (2*n* = 2*x* = 26)], was used to prepare the pachytene chromosomes and extended DNA fibers. CRIGA-1 is a highly inbred line developed by seven generations of bulk selfing methods and then plus six generations of single seed descent method from a cultivar, Shixiya-1, which was developed and cultivated in the middle of the 20^th^ century in China. The genetic stock was developed and maintained by our laboratory.

### DNA probes and labeling

The *Arabidopsis*-type telomeric repeats were amplified according to Ijdo et al. [50] and were labeled with biotin by nick translation. The 45S rDNA derived from *Arabidopsis thaliana* were kindly provided by Professor Yunchun Song, Wuhan University, China, and were labeled with biotin by nick translation. The genomic DNA of *Gossypium arboreum* was used as DNA probe and labeled with digoxigenin-11-dUTP by nick translation.

### Preparation of pachytene chromosomes

For preparation of cotton meiotic pachytene chromosomes, the significant modified method of Goel et al. [51] was used. Young floral buds, about 5∼6 mm long, were selected for meiotic chromosome preparation. The appropriate meiotic stage of development was determined. Anthers from a bud were squashed in 45% acetic acid on a slide and checked under a phase microscope. The buds with pollen mother cells (PMCs) in prophase I were fixed directly in Carnoy's solution for 30 minutes and washed twice in deionized water in Petri dish (5 cm in diameter) before anthers were removed from the bud soaked in 30 mmol•L^−1^ citrate buffer (pH 4.5) for 10 minutes. Dissected anthers were cut at the apex and squeezed with a surgical knife to extrude the PMCs into 1 mL of 30 mmol•L^−1^ citrate buffer (pH 4.5). PMCs were transferred into a 1.5 mL microcentrifuge tube. Digestion was carried out in 50 µL of enzyme mixture containing 4% (w/v) cellulase R-10 (Sigma), 0.5% (w/v) pectolyaseY-23 (Solarbio) and 1% (w/v) cytohelicase (Dingguo) at 37°C for 4 hours. Digested PMCs were collected by centrifugation at 780 rpm for 5 minutes at room temperature. The supernatant was removed and PMCs were resuspended in a solution containing 60% acetic acid and enzyme mixture (v/v, 1∶3) (above-mentioned), incubated for 3 minutes at room temperature. PMCs were again collected by centrifuging and the sediment was resuspended in 50 µL of 60% acetic acid. The PMCs suspension was transferred onto grease-free slides and incubated at 50°C for 1 minute to clear cytoplasm before a rim of ice-cold Carnoy's solution (20 µL) was put onto the slide around the PMCs suspension at room temperature. After 50 µL Carnoy's solution was dropped and mixed with the PMCs suspension, the chromosomes were spread on the slide and left to dry for 20 minutes at room temperature. Then the slide was immersed in absolute ethanol and left to dry. The slides could be used directly for the *in situ* hybridization or stored at −20°C for several months.

### Isolation of nuclei and preparation of DNA fibers

Nuclei were prepared according to Li et al. [52] with some modifications. One gram of cotyledons, which were germinated in dark moisture chamber at 37°C for one week, were collected and chopped with a sharp sterile scalpel in a Petri dish (5 cm in diameter) that contained 12.5 mL of ice-cold nucleus isolation buffer (0.01 mol•L^−1^ MgSO_4_, 0.05 mol•L^−1^KCl, 0.005 mol•L^−1^ HEPES, 1 mg•mL^−1^ dithiothreitol, 0.25% Triton X-100 and 2% PVP40) before sequentially filtered through a 100 µm, 50 µm, and 30 µm nylon mesh. The filtrates were centrifuged at 20,800 rpm for 1 minute and the supernatant was discarded. All the operations were performed on ice. The precipitation was resuspended in a 125 µL mixture containing nucleus isolation buffer and pure glycerol (1∶1). The nucleus concentration can be checked by staining with DAPI and observe under a fluorescence microscope (Leica MRA, Germany).

DNA fibers were prepared according to the modified method of Fransz et al. [7]. For a short period of time 2 µL of the suspension was deposited as a line on one end of a slide treated with poly-L-lysine and air dried for 5 to 10 minutes at room temperature. The 30 µL nucleus lysis buffer (0.5% sodium dodecylsulfate, 5 mmol•L^−1^ ethylenediaminetetraacetic acid, 100 mmol•L^−1^ Tris, pH 7.0) was added to the nuclei and incubated at room temperature for 9 minutes. DNA fibers were dragged and extended slowly and smoothly with a clean coverslip edge just ahead of the surface of the solution, followed by air drying for 10 minutes and fixing in Carnoy's solution for 2 minutes. Finally, slides were baked at 60°C for 30 minutes. Prepared slides could be used immediately or stored at −20°C for 6 to 10 months for later use.

### Fluorescence *in situ* hybridization and detection

The FISH of pachytene chromosomes was performed according to the modified method of Franz et al. [7]. The slides were pretreated with 100 mg•mL^−1^ DNase-free RNase in 2× SSC at 37°C for 1 hour and then washed three times in 1× PBS for five minutes each. The 20 µL of a hybridization mixture containing 50% formamide, 2× SSC, 10% sodium dextran sulphate, 50 mmol•L^−1^ phosphate buffer (pH 7.0), 1∼2 ng•µL^−1^ DNA probe, and 50∼100 ng•µL^−1^ salmon sperm DNA was used on each slide. *In situ* hybridization was performed at 37°C overnight, followed by several post-hybridization washes for 3×5 minutes in 50% formamide at 37°C, 3 to 5 minutes in 2× SSC at room temperature, and 3 to 5 minutes in 0.2× SSC at room temperature.

FISH on DNA fibers was performed using the modified method of Li et al. [52]. The slides were pretreated with 100 mg•mL^−1^ DNase-free RNase in 2× SSC at 37°C for 30 minutes and then washed three times in 1× PBS for 3 minutes each. Probes and DNA fibers were denatured separately and 30 µL of the hybridization mixture was added to the slide and incubated at 37°C overnight, followed by post-hybridization washes for 3×5 minutes in 50% formamide, 3×5 minutes in 2× SSC at room temperature, and 5 minutes in 1× 4T (4× SSC, 0.05% Tween 20). Then, the slide was incubated in 4 M (4× SSC, 5% defatted milk) at 37°C for 30 minutes, followed by washing in 1× 4T for 2 minutes.

All of the slides were sealed with 5% BSA at 37°C for 30 minutes and covered with avidin-fluorescein (5 ng•µL^−1^) or rhodamine-conjugated anti-digoxigenin antibodies (5 ng•µL^−1^) (Roche, Germany) at 37°C for 1 hour. The slides were washed with 1× PBS for 3×5 minutes, counterstained with 1 µg•mL^−1^ 4′, 6-diamidino-2-2phenylin-dole (DAPI, Sigma), then were washed again with 1× PBS for 3×3 minutes, and mounted in 10% Vectashield antifade (Vector, USA).

The hybridization signals were observed under a fluorescence microscope (Zeiss Axioskop 2, Germany). Images were captured by a charge-coupled device (CCD) system and the FISH images were further processed using Adobe Photoshop 7.0.

## Results and Discussions

**Figure 1:**
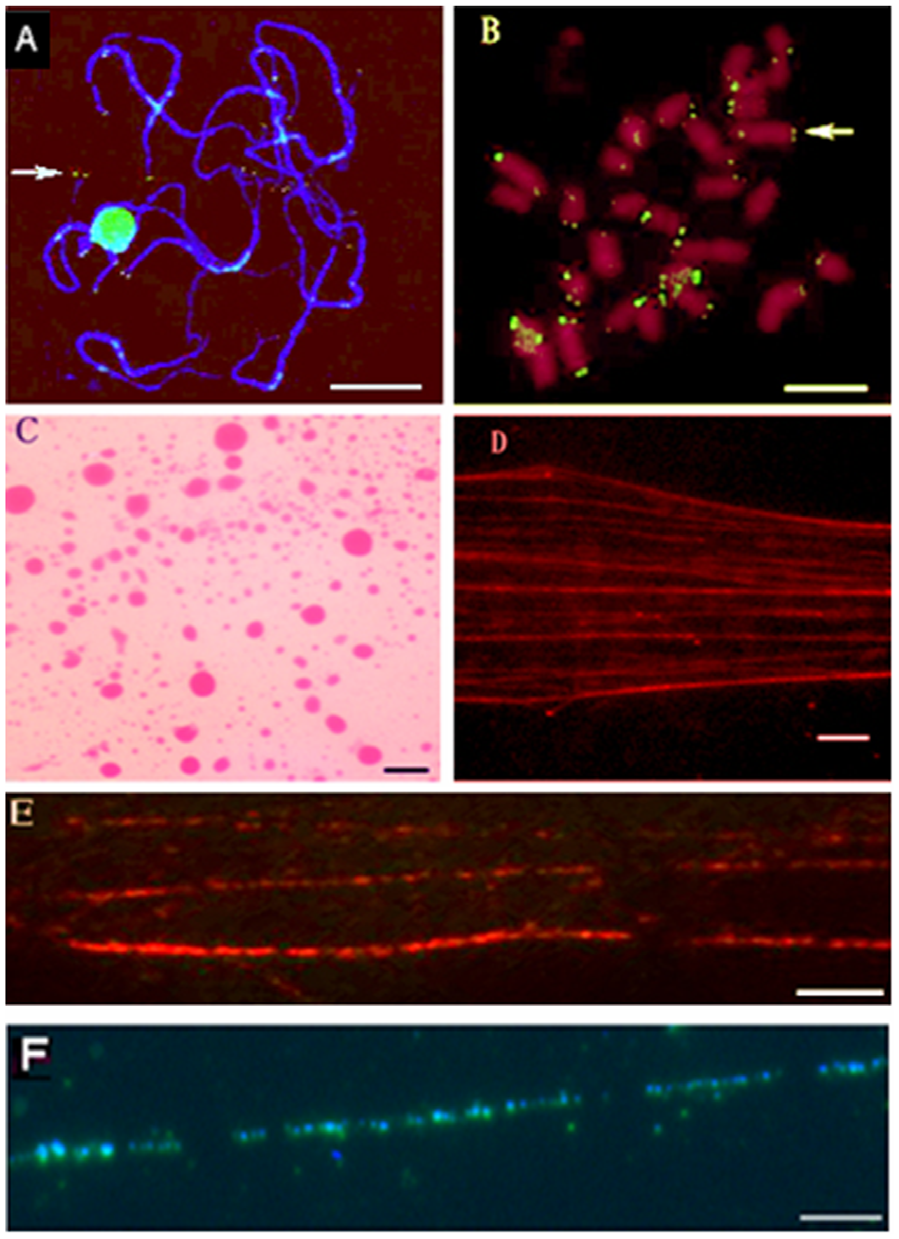
Fluorescence *in situ* hybridization patterns on pachytene chromosomes and DNA fibers of diploid cotton, *Gossypium arboreum*. **A**. FISH of telomeres on pachytene chromosomes. The *Arabidopsis*-type telomere probe was labeled with biotin and detected with avidin-fluorescein. The arrow indicates the separation of bivalents on the end of pachytene chromosomes, brilliant blue is heterochromatin and dark-blue is euchromatin. **B**. FISH of telomeres on metaphase chromosomes. The telomere probe was labeled with biotin and detected with avidin-fluorescein. The arrow indicates the telomere signal. **C**. Nuclei prepared with the chopping method, the big dots are nuclei with partly cytoplasm. **D**. The extended cotton DNA fibers prepared from nuclei. **E**. Cotton DNA fibers hybridized with genome DNA probes labeled with rhodamine-conjugated and detected with anti-digoxigenin antibody. **F**. Cotton DNA fibers hybridized with 45S rDNA probes labeled with biotin and detected with avidin-fluorescein. The chromosomes in A-F were counterstained with DAPI. (A, B Bars = 5 µm; C, D, E, F Bars = 10 µm).

The technique we have developed for preparing pachytene chromosomes from cotton can be used to obtain well-differentiated chromosomes with less entanglement and fracture, and clear background. As shown in figure 1A, there is a set of pachytene chromosomes with 13 extended bivalents, each of them represents fully paired homologous chromosomes. The 13 bivalents exhibit well-differentiated patterns of eu- and hetero- chromatin, as well as the relative position of centromere, allowing unambiguous identification of all 13 bivalents. Cotton is a malvaceous species, which contains high levels of secondary compounds, such as polysaccharides, phenols, etc [43]–[45]. Moreover, the large number and length of pachytene chromosomes can easily twist together, and are difficult to separate and spread [48], [49]. Another difficulty in preparing pachytene chromosomes of cotton is the hard cell wall which prevents the release of chromosome. Our initial experiments primarily focused on improving the technique to achieve optimal spreading of well-differentiated cotton pachytene chromosomes suitable for FISH. The improved method has several key steps. Firstly, enzyme digestion was done directly on PMCs to completely break down the hard cell wall. With other method, the enzyme digestion was done on anthers in buds [45], [48], in which enzyme concentration and digestion time are not easy to control. Secondly, before being incubated in 60% acetic acid, PMCs were incubated in the solution of 60% acetic acid and enzyme mixture at room temperature for 3 minutes to clear cytoplasm. Compared with single treatment with 60% acetic acid [45], the cell contents can be removed completely. Thirdly, after a rim of ice-cold Carnoy's solution was put onto the slide around the PMCs suspension at room temperature, another 50 µL ice-cold Carnoy's solution was dropped and mixed with the PMCs suspension, the chromosomes were fully stretched on the slide by the help of Carnoy's solution (Fig. 1A). The placed coverslip and nitrogen removed procedures were omitted in order to prevent chromosome loss, entanglement and fracture [45], [48], [49].

In this study, more than 20,000 nuclei per gram of fresh etiolated young cotyledons were obtained at 20,800 rpm for 1 minute, with less debris (Fig. 1C). The key modification of this method was that the routine liquid nitrogen grinding of leaves [18], [53]–[55] was replaced by chopping fresh etiolated young cotyledons with blade in ice-cold nucleus isolation buffer. With the liquid nitrogen method, over- or under-grinding of leaves occurs more frequently, and DNA fibers with the desired quality are not obtained easily. In comparison with nitrogen grinding method, the chopping method is much easier to control, the nuclei are easy released, and fewer nuclei are destroyed. Because of having less chloroplasts and other cytoplasm substitutes [12], [45], [55], the fresh etiolated young cotyledons germinated in dark, humid chamber at 37°C for one week were used for preparation. The PVP40 in the isolated solution buffer can inhibit the high levels of phenolic compounds in cotton cell [55]. Intact and cleaner nuclei were obtained by the use of fresh etiolated young cotyledons and the treatment of PVP40 in the isolated solution buffer [Fig. 1C]. To examine the effects of the centrifuge time on the quantity and quality of nuclei recovery, different centrifuge times of 20 s, 40 s, 60 s, 80 s, and longer times were tested at a high speed (20,800 rpm). The best result occurred at 60 s centrifugation with less debris [Fig. 1C]. There are not enough nuclei acquired at centrifuge times of 20 s and 40 s (data not shown). Although more nuclei could be obtained at longer centrifuge times, there would be more debris (data not shown). We chose to drag with a coverslip edge just ahead of the solution to spread chromosomes on the slide (we called Liquid Drainage Method) in order to avoid the accumulation, twist and fracture of DNA fibers. Dragging should be carried out slowly and smoothly along the slide. The method of tilting slide to let the solution flow downward and spread the chromosomes is adopted in plant [56], but the twist and accumulation of DNA fibers can't be avoided. With the method of spreading DNA fibers with coverslip on slide [7], [27], [33], [45], [52], the degree of the strength is difficult to control, and easily lead to DNA fibers entanglement and fracture. In comparison with above two methods, a stretched pattern of long and thin parallel threads of cotton DNA fibers were exhibited with our method (Fig. 1D, 1E). Isolation of the nuclei takes 3 h with the routine nitrogen grinding method [7], [27], [53], [55], in contrast, the chopping method is rapid and takes only 30 minutes for the entire process from leaf chopping to extension of DNA fibers. The procedure is also safe because nonpoisonous dithiothreitol is used instead of poisonous mercaptoethanol. The probability of success in isolating nuclei for DNA fiber preparation is almost 100% for the cotton species tested with this method (Fig. 1D).

In order to examine the quality of the prepared pachytene chromosomes, FISH was conducted using biotin-labeled *Arabidopsis*-type telomere probes. The 13 pachytene bivalent ends with green telomere signals were observed as shown in figure 1A. The signal intensity varied obviously among the different chromosomes in meiosis pachytene and mitotic metaphase. (Fig. 1A, 1B), which suggested a considerable variation in sequence length of this satellite repeat among the chromosomes. The pachytene bivalents exhibited a distribution of distal telomere signals similar to those of the metaphase chromosomes. The bright telomere signals on the ends of well-differentiated pachytene chromosomes and the clear characteristic differentiation of euchromatin and heterochromatin segments of cotton pachytene bivalents allows further identification of all 13 bivalents in a high-resolution karyotype analysis. FISH on well- differentiated pachytene chromosomes is considered a useful method, which provides an excellent way to develop a physical cytological map for species with small chromosomes [57]. The results elucidated that the pachytene chromosomes obtained by our technique was suitable for FISH and it would offer considerable potential for producing high-resolution physical maps of cotton like rice [25] or sorghum [58].

To examine the integrity and length of single extended DNA fibers and their suitability for hybridization, DNA fiber preparations were hybridized with genomic DNA and 45S rDNA as probes. The signals were observed linear or nearly linear stretches of beads on-a-string fluorescence signals (Fig. 1E and 1F), which is nearly the same as that observed by the classical method [18]. The typical discontinuous pattern of DNA fiber in FISH can be caused by various factors, such as loss and inaccessibility of target DNA due to either *in situ* renaturation or attachment to the glass substrate, as well as suppression of repeat sequences [59]–[62]. It was concluded that the extended DNA fibers generated with this new method can be used to map and analyze large repeated sequences and genomic DNA libraries, such as BAC and YAC clones.
